# Head-to-head comparison of clustering methods for heterogeneous data: a simulation-driven benchmark

**DOI:** 10.1038/s41598-021-83340-8

**Published:** 2021-02-18

**Authors:** Gregoire Preud’homme, Kevin Duarte, Kevin Dalleau, Claire Lacomblez, Emmanuel Bresso, Malika Smaïl-Tabbone, Miguel Couceiro, Marie-Dominique Devignes, Masatake Kobayashi, Olivier Huttin, João Pedro Ferreira, Faiez Zannad, Patrick Rossignol, Nicolas Girerd

**Affiliations:** 1 Centre d’Investigations Cliniques Plurithématique 1433, INSERM 1116, CHRU de Nancy, Université de Lorraine, Nancy, France; 2 F-CRIN INI-CRCT Cardiovascular and Renal Clinical Trialists Network, Nancy, France; 3 CNRS, Inria Nancy Grand-Est, LORIA, UMR 7503, Université de Lorraine, Vandoeuvre-lès-Nancy, France; 4 Centre d’Investigation Clinique Pierre Drouin -INSERM - CHRU de Nancy, Institut Lorrain du cœur Et Des Vaisseaux Louis Mathieu, 4, Rue du Morvan, 54500 Vandœuvre-Lès-Nancy, France

**Keywords:** Computational biology and bioinformatics, Functional clustering, Machine learning

## Abstract

The choice of the most appropriate unsupervised machine-learning method for “heterogeneous” or “mixed” data, i.e. with both continuous and categorical variables, can be challenging. Our aim was to examine the performance of various clustering strategies for mixed data using both simulated and real-life data. We conducted a benchmark analysis of “ready-to-use” tools in R comparing 4 model-based (Kamila algorithm, Latent Class Analysis, Latent Class Model [LCM] and Clustering by Mixture Modeling) and 5 distance/dissimilarity-based (Gower distance or Unsupervised Extra Trees dissimilarity followed by hierarchical clustering or Partitioning Around Medoids, K-prototypes) clustering methods. Clustering performances were assessed by Adjusted Rand Index (ARI) on 1000 generated virtual populations consisting of mixed variables using 7 scenarios with varying population sizes, number of clusters, number of continuous and categorical variables, proportions of relevant (non-noisy) variables and degree of variable relevance (low, mild, high). Clustering methods were then applied on the EPHESUS randomized clinical trial data (a heart failure trial evaluating the effect of eplerenone) allowing to illustrate the differences between different clustering techniques. The simulations revealed the dominance of K-prototypes, Kamila and LCM models over all other methods. Overall, methods using dissimilarity matrices in classical algorithms such as Partitioning Around Medoids and Hierarchical Clustering had a lower ARI compared to model-based methods in all scenarios. When applying clustering methods to a real-life clinical dataset, LCM showed promising results with regard to differences in (1) clinical profiles across clusters, (2) prognostic performance (highest C-index) and (3) identification of patient subgroups with substantial treatment benefit. The present findings suggest key differences in clustering performance between the tested algorithms (limited to tools readily available in R). In most of the tested scenarios, model-based methods (in particular the Kamila and LCM packages) and K-prototypes typically performed best in the setting of heterogeneous data.

## Introduction

Cluster analysis aims to partition unlabeled data into homogeneous groups, such that two instances are similar if they belong to the same cluster, and dissimilar otherwise. Although this unsupervised machine-learning task is often considered in the context of either continuous or categorical datasets, this task remains challenging when dealing with “heterogeneous” or “mixed” data, *i.e.* with both types of variables. As previously emphasized, clustering of mixed data is challenging because it is difficult to directly apply mathematical operations to both types of feature variables^1^. One of the main issues arising in the framework of mixed data clustering is thus the choice of the most appropriate distance or model to simultaneously process both data types. Indeed, clinical research usually relies on heterogeneous data: clinical datasets typically include a mix of variables related to clinical history (usually categorical variables), general/anthropometric data (usually continuous variables such as age and body mass index), physical examination (both categorical and ordinal variables) and laboratory or imaging findings (often continuous variables). Note that among laboratory variables, omics data are increasingly available today. Such heterogeneity urges for ways to guide users and clinical practitioners in choosing appropriate clustering approaches for heterogeneous clinical datasets in order to achieve efficient phenomapping of patients in various clinical settings.

Discretization and dummy-coding are some of the simple and intuitive solutions to obtain a homogeneous dataset containing only categorical data on which classical techniques can be applied. However, this approach may introduce distortion in the original data and may consequently lead to increased bias^2^. Fortunately, a wide range of clustering algorithms has been specifically developed to deal with mixed data. A detailed taxonomy of available methods has been reported recently by Ahmad and Khan ^1^. Nevertheless, the end-user may be bewildered when choosing one of these techniques as there is no clear guidance for choosing the most appropriate technique in a given context. To our knowledge, few benchmark studies have examined the performance of clustering strategies for mixed type variables on both real and simulated data^3^. Moreover, only a few of the available techniques have been tested in previous benchmark attempts. In addition, an external assessment of available techniques, by a group not directly involved in their development, may further strengthen the generalizability of the results. In fact, a better understanding of the strengths and weaknesses of each clustering strategy may help to clarify the lack of reproducibility and generalization sometimes observed in the setting of mixed data clustering.

The present study aims to assess the performance of clustering strategies for mixed data in both simulated and real case scenarios. In the first group of scenarios, virtual populations with available mixed variables were generated on which a benchmark of clustering techniques was conducted. The same techniques were subsequently applied to a real-life dataset from the EPHESUS randomized clinical trial^4^ to illustrate the importance of choosing the appropriate clustering technique. As our focus was to test tools readily/easily available to clinical researchers, we therefore restricted our analysis to “off-the-shelf” tools readily available from the R software (R Core Team), that cover only a portion of all available methods for clustering heterogeneous data.

## Methods

### Clustering algorithms

#### Design questions

From a formal point of view, three design questions must be addressed in the specific setting of mixed data clustering. The first question (Q1) is how to calculate similarities/distances for categorical and numeric data when using distance-based algorithms, or how to transform the data for model-based methods. The second question (Q2) is related to the methodology to merge numerical and categorical parts. The last question (Q3) is the choice of the algorithm that will be used to build optimal clusters.

As mentioned above, to facilitate the evaluation process, we selected only clustering algorithms either already present or easily implementable in the R software (R version 3.6.3, R Core Team). Thus, and due to computing time load and lack of availability of some of them, only a limited number of representative techniques were retained for this study. The selected algorithms are described in Table 1 according to the three design questions relevant for heterogeneous data. Algorithms are grouped as distance-based or model-based. In the first group, the dissimilarities (Q1) used by the various algorithms are different between numeric and categorical data except for the UET distance (see below "Unsupervised extra trees dissimilarity (UET)" section). Merging (Q2) is therefore not required with UET distance. Two optimization algorithms (Q3): K-medoids or hierarchical ascendant clustering (HAC) using Ward aggregation measure (see below "Partitioning around medoids (PAM)" and "Ascendant hierarchical clustering (HC)" sections respectively) have been used with Gower and UET dissimilarity matrices, whereas the K-prototypes (see "K-prototypes (kproto)" section) uses K-means. For most model-based methods, the distributions of both numeric and categorical variables are transformed (Q1) into probabilities, except for Kamila which uses Euclidean distance to handle numeric variables and probabilities for categorical ones (see "Kamila" section). Therefore, Kamila needs to set up an ensemble-like approach to merge (Q2) both types of data, using both K-means and Expectation Maximization (EM) as optimization algorithms (Q3). The three other model-based methods do not need any merging procedure as both types of variables are included in a unique probabilistic model and use EM algorithms with specific variants (see below "Clustering by mixture modeling (Mixmod)" to "Latent class analysis (LCA)" sections).

**Table 1:** Description of selected methods with regards to design questions related to (1) similarities/distances or data transformation, (2) methodology to merge numerical and categorical parts and (3) algorithm choice.

Clustering method	Q1: Distance or transformation		Q2: Merge mode	Q3: Optimization algorithm
	Numeric	Categorical		
**Distance-based methods**				
PAM^a^	Normalized difference	Hamming	Gower^b^	K-medoids
	UET^c^	UET^c^	NA	K-medoids
Ascendant hierarchical clustering^d^	Normalized difference	Hamming	Gower^b^	HAC + Ward link
	UET^c^	UET^c^	NA	HAC + ward link
Kproto^e^	Euclidean	Hamming	Weighted sum	Kmeans
**Model-based methods**				
Kamila^f^	Euclidean	Probabilities	ensemble-like approach	K-means and EM
LCA^g^	Discretisation and probabilities	Probabilities	NA	EM and Newton–Raphson
LCM^h^	Probabilities	Probabilities	NA	EM + feature selection
Mixmod^i^	Probabilities	Probabilities	NA	EM

NA, Not applicable; EM, Expectation Maximization ; PAM, Partitioning Around Medoids; HAC, Hierarchical Ascendant Clustering; UET, Unsupervised Extra Trees dissimilarity.

^a^clustMixType package (PAM function): https://cran.r-project.org/web/packages/clustMixType.

^b^Cluster package (daisy function): https://cran.r-project.org/web/packages/cluster.

^c^Yet unpublished UET package, available at https://gitlab.inria.fr/kdalleau/uetcpp, build_randomized_tree_and_get_sim function.

^d^Stats (R-base package, hclust function): https://stat.ethz.ch/R-manual//R-devel/library/stats/html/00Index.html.

^e^clustMixType package (kproto function); see ^a^.

^f^Kamila package (kamila function): https://cran.r-project.org/web/packages/kamila.

^g^poLCA package (poLCA function): https://cran.r-project.org/web/packages/poLCA.

^h^VarSelLCM package (VarSelCluster function): https://cran.r-project.org/web/packages/VarSelLCM.

^i^Rmixmod package (mixmodCluster function): https://cran.r-project.org/web/packages/Rmixmod.

The following sections aims at briefly describing all the clustering methods used in this study and their underlying mechanisms. The following notations are used throughout this section: $G$ is the number of clusters, $N$ the size of the population, $p$ the total number of variables.

#### Distance-based methods

This family of methods relies exclusively on explicit distances or dissimilarities between individuals. Some algorithms such as Partitioning Around Medoids (PAM) or Hierarchical Ascendant Clustering (HAC) can take any dissimilarity matrix as an input, whereas K-prototypes rather build their own distance. Note that in the present analysis, by misuse of language, the term “distance” sometimes means “dissimilarity” as some measures do not necessarily verify the triangular inequality.

##### Gower-based dissimilarity

For two observations $x$ and $y$, the Gower^5^ (1971) similarity coefficient is represented as:

$$S\left(x,y\right)=\frac{1}{m}\left(\sum\limits_{j=1}^{q}\left(1-\frac{\left|{x}_{j}-{y}_{j}\right|}{Range\left(j\right)}\right)+\sum \limits_{j=q+1}^{p}s\left({x}_{j},{y}_{j}\right)\right)$$

where $s\left({x}_{j},{y}_{j}\right)$ equals 1 if ${x}_{j}={y}_{j}$ and 0 otherwise, and Range(j) represents the absolute difference between extreme values of the j-th variable.

The first term of the right part is the similarity on the continuous variables, while the second term deals with categorical variables. By dividing the difference |*x*_*j*_*—y*_*j*_| by the range of variable j, both coefficients for numeric and categorical variables are included in the interval $[\mathrm{0,1}]$. The dissimilarity matrix is then comprised of the dissimilarity coefficients calculated between each pair of observations. In the *daisy* function from the *cluster* R package, the transformation $1-S\left(x,y\right)$ is used, although $\sqrt{1-S\left(x,y\right)}$ was initially proposed by Gower^5^ (1971). In this implementation of the Gower-based dissimilarity, as emphasized in Table 1, the categorical part of the data is actually handled by the Hamming distance.

##### Unsupervised extra trees dissimilarity (UET)

This recently published^6^ method for computing dissimilarity measurements relies on the principle of decision trees. Unlike traditional approaches, UET does not require a target variable to assess the homogeneity of the final nodes and to perform the respective splits. Rather, at each step, the method samples a variable without replacement and then samples a threshold among the values of this variable. The population of the parent node is divided into two child nodes, according to the value of the observation relative to the threshold (lower/greater than for continuous variables, equal/different for categorical variables). The tree growth is halted when there is no remaining variable or attribute, or if the current node has a size lower than a certain value, the *smoothing parameter.* This parameter controls the depth of each tree. For each tree built, the similarity matrix is updated: for each pair of observations, if they are in the same terminal node, their similarity is incremented by 1. The algorithm is repeated M times so that each pairwise similarity is divided by M at the end. The similarities are then converted into dissimilarities using the formula $\sqrt{1-S\left(x,y\right)}$.

Unlike other dissimilarities such as the Euclidean distance for k-means clustering, the UET dissimilarity does not require scaling the data displaying different magnitudes, thus preserving the original structure. This approach is moreover functional for homogeneous as well as heterogeneous data and is robust to outliers and noise^6^.

There is presently no official R package for this algorithm, but the source code and the instructions for its installation can be found on Gitlab (https://gitlab.inria.fr/kdalleau/uetcpp).

Each of the previous dissimilarity matrices can then be incorporated into one of the two following clustering algorithms: Partitioning Around Medoids (PAM) and Hierarchical Clustering (HC).

##### Partitioning around medoids (PAM)

The PAM method^7^ builds a partition by affecting observations to the closest “medoid”, i.e. the best representative subject of its cluster. The algorithm is composed of two steps: one for building the current clustering similarly to the K-means (BUILD phase), and another to improve the partition toward a local optimum (SWAP phase).

The minimization criteria is the Total Deviation (TD):

$$TD=\sum \limits_{g=1}^{G}\sum \limits_{{x}_{j}\in {C}_{g}}d\left({x}_{j},{m}_{g}\right)$$

where $\left({m}_{1},...,{m}_{G}\right)$ are the medoids,$\left({C}_{1},...,{C}_{G}\right)$ the respective clusters they represent, and $d\left({x}_{j},{m}_{g}\right)$ the dissimilarity between the subject ${x}_{j}$ and the medoid of the cluster ${C}_{g}$.

The BUILD phase finds the first medoid which minimizes the total deviation, i.e. with the smallest dissimilarity to all other subjects. The remaining $G-1$ medoids are then successively found by maximizing the reduction of the TD.

The SWAP phase subsequently improves the existing partition by considering all possible “swaps” of the G medoids with the non-medoids. The swaps which reduce TD the most are applied, and the process is repeated until no further improvement is found. This method is implemented in the *pam* function of the *cluster* R package.

##### Ascendant hierarchical clustering (HC)

This well-known clustering method begins with N clusters (one per subject), then at each step aggregates the two closest clusters until only one remain. The successive fusions are represented on a dendrogram to facilitate the a posteriori choice of an optimal number of clusters. In general, the best partition is the one preceding the first sizeable increase in intra-cluster variance.

Let us suppose that at a particular aggregation step, clusters ${C}_{i}$ and ${C}_{j}$ are the next to be merged. To determine the distance of the merged cluster ${C}_{i}\cup {C}_{j}$ with any other cluster ${C}_{k}$, the dissimilarity matrix must be updated by one aggregation method belonging to the Lance-Williams algorithm family:

$$d\left({C}_{i}\cup {C}_{j},{C}_{k}\right)=\alpha d\left({C}_{i},{C}_{k}\right)+\beta d\left({C}_{j},{C}_{k}\right)-\eta d\left({C}_{i},{C}_{j}\right)$$

The coefficients α, β and η are dependent on the aggregation method. These methods for computing distances between clusters are called linkage criteria. For the present benchmark, Ward’s algorithm^8^ was chosen, which aims at minimizing the increase in intra-cluster variance at each binary fusion, such that convex and compact clusters are more likely to be formed. With the Ward’s aggregation method, the formula becomes:

$$d\left({C}_{i}\cup {C}_{j},{C}_{k}\right)=\frac{{n}_{i}+{n}_{k}}{{n}_{i}+{n}_{j}+{n}_{k}}d\left({C}_{i},{C}_{k}\right)+\frac{{n}_{j}+{n}_{k}}{{n}_{i}+{n}_{j}+{n}_{k}}d\left({C}_{j},{C}_{k}\right)-\frac{{n}_{k}}{{n}_{i}+{n}_{j}+{n}_{k}}d\left({C}_{i},{C}_{j}\right)$$

with ${n}_{i}$, ${n}_{j}$ and ${n}_{k}$ representing the respective sample sizes of ${C}_{i}$,${C}_{j}$ and ${C}_{k}$.

Ward’s algorithm is implemented in the *hclust* function of the *stats* R package, when *method* = *“ward.D2*” is selected.

##### K-prototypes (Kproto)

The K-prototypes algorithm^9^ defines $G$ virtual individuals (or prototypes) as the centers of the groups, built from the means by group for numeric variables, and modes by group for categorical variables. The distance between two subjects X and Y is then defined as: ${d}_{2}\left(X,Y\right)={\sum }_{j=1}^{q}{\left({x}_{j}-{y}_{j}\right)}^{2}+\gamma {\sum }_{j=q+1}^{p}\delta \left({x}_{j},{y}_{j}\right)$

where the first term is the squared Euclidean distance measurement for the continuous variables and the second term is the Hamming distance^10^ (1950). The weight γ is used to avoid favoring either type of attribute. It can be specified by the user or estimated via a combined variance of the data.

The minimization criteria is the total sum of distances (TSD) between the subjects and the prototype of the class ${b}_{g}$ to which they belong: $TSD={\sum }_{g=1}^{G}{\sum }_{x\in {C}_{g}}\left({\sum }_{j=1}^{q}{\left({x}_{j}-{b}_{g,j}\right)}^{2}+\gamma {\sum }_{j=q+1}^{p}\delta \left({x}_{j},{b}_{g,j}\right)\right)$

In practice, the algorithm is very similar to the k-means: initial G prototypes are selected as temporary centers of the clusters, then each subject is allocated to the closest prototypes. When all subjects are allocated, the prototypes are updated to represent their optimal class. The subjects are then reallocated to the updated prototypes if needed, and the process is repeated until the partition is stable. This algorithm can be found in the (*kproto, Kproto)* function of the *clustMixType* R package.

#### Model-based methods

##### Kamila

The Kamila algorithm^2^ is a model-based adaptation of the k-means for managing heterogeneous datasets. The sample of continuous variables is assumed to follow a mixture distribution with arbitrary spherical clusters (where the density of the data is only dependent on the distance to the center of the distribution). This assumption is less restrictive than those from Mixmod or LCM (see below). Categorical variables are supposed to be sampled from a mixture of multinomial variables. Factors are also assumed to be conditionally independent given the clusters to which they belong.

The Kamila algorithm begins with a set of centroids for the continuous variables and a set of parameters for the categorical variables. For continuous variables, the Euclidean distance with the closest centroid is computed. This set of N minimal distances is used to estimate the mixture distribution of continuous variables. For categorical variables, the probabilities of observing the data given the cluster are computed.

The log-likelihood of the sum of these two components is then used to find the most appropriate cluster for each subject. Based on this temporary partition, the centroids and the parameters are updated to best represent the clusters.

These steps are repeated until the clusters are stable. Finally, multiple runs of this process are performed with different initializations, and the partition maximizing the sum of the best final likelihoods is retained.

The R package *kamila* is a direct implementation of this technique by its authors.

##### Clustering by mixture modeling (Mixmod)

Clustering by mixture modeling was proposed a number of years ago^11^, although powerful computers are needed to realize its full potential. Nowadays, many R packages implement mixture models such as *clustMD* or *fpc,* although we preferred the *Rmixmod*^12^ package for its rich parametrization and cross-platform implementation.

Mixture models assume that continuous variables follow a multivariate normal distribution whereas categorical variables follow a multivariate multinomial distribution. For an observation ${x}_{i}$, the probability distribution function is defined as:

$$f\left({x}_{i}|\theta \right)=\sum\limits_{g=1}^{G}{\tau }_{g}h\left({x}_{i}|{\alpha }_{g}\right)$$

where $h\left({x}_{i}|{\alpha }_{g}\right)$ is the distribution function for cluster $g$, with parameters ${\alpha }_{g}$. For example, if $h$ is defined as a multivariate normal distribution, ${\alpha }_{g}$ would be the mean vector ${\mu }_{g}$ and the variance–covariance matrix ${\Sigma }_{g}$. The mixing proportions ${\tau }_{g}\in ]\mathrm{0,1}[$ sum to 1, and thus describe the expected size of each cluster. The set of parameters to be determined is $\theta =\left({\tau }_{1},...,{\tau }_{g},{\alpha }_{1},...,{\alpha }_{g}\right)$.

Given the parameters and the data, the probability for a subject $i$ to be classified into cluster $g$ is:

$${t}_{ig}\left(\theta \right)=\frac{{\tau }_{g}h\left({x}_{i}|{\alpha }_{g}\right)}{f\left({x}_{i}|\theta \right)}$$

Thus, for each individual $i$, we define the $G\times 1$ dummy vector ${z}_{ig}\left(\theta \right)$ containing 1 where ${t}_{ig}\left(\theta \right)$ is maximum, and 0 elsewhere. These ${z}_{ig}$ are then introduced into the completed log-likelihood of the observed data:

$${L}_{c}\left(\theta ,z\right)=\sum\limits_{i=1}^{N}\sum\limits_{g=1}^{G}{z}_{ig}ln\left({\tau }_{g}h\left({x}_{i}|{\alpha }_{g}\right)\right)$$

Following an Expectation–Maximization (EM) framework, the set of parameters $\theta$ is computed such that the log-likelihood is maximized. The ${t}_{ig}\left(\theta \right)$ are then updated and so forth until convergence is reached.

The crucial portion of this process relies on the choice of the model for the data within a specific cluster, i.e. the distribution function $h$. Several models are available with different levels of constraints.

For continuous variables, the variance–covariance matrices are assumed to be diagonal. The user can decide to set all cluster volumes equal, and/or all intra-variances equal, which yields 4 possible models.

With regard to categorical variables, a re-parametrization allows an interpretation similar to the center and the variance matrix used for continuous data. The dispersion parameter can be chosen to be the same across clusters and/or across variables, or across levels, thereby yielding 5 possibilities.

##### Latent class model (LCM)

This method, implemented by its authors in the *VarSelLCM* R package^13^, is another type of mixture modeling quite similar to Mixmod but, in addition, it can also determine whether a variable is useful for clustering, as well as the optimal number of clusters.

If the j-th variable is relevant (i.e., its distribution differs significantly across clusters), it is labeled with ${\omega }_{j}=1$ and belongs to $\Omega$. If j is irrelevant (i.e. its distribution is similar across clusters), it is labeled with ${\omega }_{j}=0$ and belongs to $\Omega$’s complementary, i.e. ${\Omega }^{c}$. Let $\omega =\left({\omega }_{1},...,{\omega }_{p}\right)$ be the binary vector of the role of the p variables, and let $m=\left(G,\omega \right)$ be the resulting model.

For an observation ${x}_{i}$, the probability density function of the mixture distribution is:

$$f\left({x}_{i}|m,\theta \right)=\prod\limits_{j\in {\Omega }^{c}}{h}_{j}\left({x}_{ij}|{\alpha }_{1j}\right)\sum\limits_{g=1}^{G}{\tau }_{g}\prod\limits_{j\in \Omega }{h}_{j}\left({x}_{ij}|{\alpha }_{gj}\right)$$

In LCM, the variables are assumed to be independent within clusters. Similarly to Mixmod, an EM algorithm is used to determine the optimal partition.

When the selection of relevant variables is enabled, a penalization on the Bayesian Information Criterion (BIC) or the Maximum Integrated Complete-data Likelihood (MICL) is applied at the maximization step. The selection of the number of clusters is achieved by running the algorithm for each number of clusters in a specified range, and selecting the one which yields the best value of the selected criterion. These selection features were not used in the present study due to the lengthy computing time, although the user may find convenient having an all-in-one tool via this function.

##### Latent class analysis (LCA)

This clustering technique is derived from the Latent Class Regression^14^ and implemented in the *poLCA* R package ^15^. Since *poLCA* was used in Ferreira et al.^16^ on the EMPHASIS and EPHESUS studies, this technique was selected in our benchmark. LCA has the particularity of being applied to categorical data only, implying that continuous variables must be discretized. This transformation can be achieved based on percentiles in order to obtain balanced level counts, or based on practitioner knowledge such that the categories are clinically relevant. Each categorical variable is supposed to be sampled from a mixture of multinomial distributions, depending to which latent cluster the subjects belong to. Similarly to mixture modeling methods, the overall density function is used:

$$f\left({x}_{i}|\theta \right)=\sum\limits_{g=1}^{G}{\tau }_{g}h\left({x}_{i}|{\alpha }_{g}\right)$$

In this instance, the ${\alpha }_{g}$ are the sets of probabilities for each level of each categorical variable if the subject belongs to the latent cluster ${C}_{g}$. Initially, the ${\tau }_{g}$ are uniform (equal cluster sizes), and the ${\alpha }_{g}$ are randomly sampled. As in Mixmod, the ${t}_{ig}\left(\theta \right)$ are computed and used to update the ${\alpha }_{g}$ according to the Bayes theorem and the observed data. With the new probabilities of the multinomial mixture, the ${\tau }_{g}$ are updated. Finally, the new parameters allow computing the log-likelihood of the present iteration:

$$LogLik\left(x|\theta \right)=\sum\limits_{i=1}^{N}{log}_{e}\left(\sum\limits_{g=1}^{G}{\tau }_{g}h\left(x|{\alpha }_{g}\right)\right)$$

The parameter update is repeated until the maximum number of iterations is achieved, or the difference between two successive log-likelihoods is too small (1e−10). Several runs are subsequently performed to avoid finding a local optimum, and the run with the best final log-likelihood returns the resulting partition.

### Simulation framework

#### General process

To assess the performance of each previous clustering method, 1000 datasets were generated with a particular design (e.g. set of parameters), yielding continuous and categorical variables which were representative of a true known partition. Each method then yielded its predicted partition that was compared to the original partition. The agreement scores over the 1000 repetitions were then summarized and graphically represented, allowing a visual comparison of the clustering performance for each method.

#### Investigated scenarios

The datasets were simulated under various scenarios in order to approach the diversity of real-life data. These scenarios were defined by controlling the following parameters:

- The size of the population (300, 600, 1200);
- The number of clusters (2, 6, 10);
- The ratio between the number of continuous and categorical variables;
- The proportion of relevant (non-noisy) variables (20%, 50%, 90%);
- The degree of relevance of the variables (low, mild, high)

Ideally, all combinations of the different values of the parameters should be tested to avoid hidden interactions. However, due to the overwhelming computational time, it was decided to investigate one parameter at a time. By default, the size of the population was 300, the number of clusters was 6, the continuous and categorical variables were equally represented (4 each), mildly relevant (see below for the definitions) and without any fully irrelevant variables. The seven scenarios and their investigated parameters are summarized in Table 2.

**Table 2:** Parameters of the simulated populations according to each tested scenario.

	1	2	3	4	5	6	7
**General parameters**							
Population size	**300/600/1200**	300	300	300	300	300	300
Number of clusters	6	**2/6/10**	6	6	6	6	6
**Continuous variables**							
Total number	4	4	**2/4/8**	4	4	4	10
Proportion of relevant variables	100%	100%	100%	100%	100%	100%	**20%/50%/90%**
Degree of relevance	Mild	Mild	Mild	**Low/mild/high**	Mild	Mild	Mild
**Categorical variables**							
Total number	4	4	**2 / 4 / 8**	4	4	10	4
Proportion of relevant variables	100%	100%	100%	100%	100%	**20%/50%/90%**	100%
Degree of relevance	Mild	Mild	Mild	Mild	**Low/mild/high**	mild	Mild

For example, in scenario n°1, three series of 1000 datasets were generated, corresponding to respective population sizes of 300, 600 and 1200. This enabled the visualization of the impact of sample size on clustering performance.

#### Simulated datasets

Each population was created according to the following process:

Continuous variables were generated by Qiu and Joe’s method^17^, an improvement of Milligan’s method^18^. This algorithm finds a multivariate normal distribution for each cluster such that a degree of separation of each cluster with its closest neighbor is verified. The $N\times q$ covariates values are then sampled and returned with the true partition. In practice, the continuous covariates were generated by using the *genRandomClust* function from the *clustergeneration* package. This function uses the separation index as a parameter, included in the $]-2, + 2[$ interval. The larger the index, the more the clusters are separated. Therefore, this index can be deemed as a degree of relevance for continuous variables. In the scenarios presented in Table 2, the separation index values “− 0.3”, “0” and “0.3” represent a “low”, “mild” and “high” degree of relevance, respectively.

In practice, when using the Euclidean distance such as in K-prototypes, continuous data should be scaled (i.e. subtracted by the mean and divided by the standard deviation). Indeed, variables having the highest values tend to play an over-represented role when computing the distance to the closest center of the class. Although all simulated continuous variables had roughly the same scale, the datasets were scaled for precautionary reasons. For the purpose of equity, all methods utilized the same scaled dataset as input, since we were more interested in relative performance than in absolute performance.

Categorical variables were then added to the population based on the existing partition. By convenience, the number of levels per variable was equal to the number of clusters, such that a single categorical variable can theoretically predict the entire partition. At the outset, each cluster and its corresponding subjects were affected to one level of one variable. Thereafter, a fraction of the categorical variable levels was resampled within each cluster, thus introducing a controlled “noise” in the variable.

For example, in order to generate a three-level variable and to control the information carried by latter relative to a partition of 3 groups with 50 subjects each, the simulation began with the “perfect” situation: on the left panel of supplementary Fig. 1, one level corresponds to one cluster. A noise proportion of 20% was then introduced, meaning that 20% of the population had its value randomly resampled among one of the three levels (right panel of supplementary Fig. 1).

Similarly to continuous variables, the introduced proportion of noise can be considered as a degree of relevance for categorical variables. In the scenarios presented in Table 2, the “low”, “mild” and “high” degrees of relevance represent noise proportion of 95%, 85% and 75%, respectively.

Noisy variables, i.e. which do not carry information regarding the latent partition, can also be added to the dataset in order to modify the proportion of relevant variables in the dataset, as described for scenarios 6 and 7 in Table 2, leading to proportions of 1/5, 1/2 and 9/10 for a total of 10 variables for the varying type. For continuous variables, the *genRandomClust* function automatically produces the latter, while for categorical variables, a full random sampling of the levels of the new variable is sufficient.

In order to visualize the data and assess the difficulty of the clustering task, we applied t-distributed stochastic neighbor embedding (t-SNE)^19^ on the numerical values to obtain a 2D representation of the clusters. The resulting plots are presented in Supplementary Fig. 2 for all scenarios. This representation shows that the simulated clusters have regular convex shapes more or less well delineated depending on the relevance degree of the variables (scenario 4) or on the % of noise added to the data (scenario 7).

#### Parameters of the clustering methods

The various clustering methods presented in "Clustering algorithms" section feature parameters which directly influence the resulting partition. Since thousands of datasets were generated for each of the seven scenarios, it was not possible to fine-tune the parameters individually. Thus, default parameters were chosen following the guidelines of the documentation, and the same parameters were implemented for every dataset. Particular attention was given to the number of initializations (e.g. for UET, K-Proto) which represents a trade-off between the robustness of the partition and computation time.

Typically, the true number of clusters is unknown and is determined a posteriori, either according to an objective criterion or according to practitioner knowledge. In this simulation framework, allowing the methods to estimate the optimal number of clusters would be hard to implement as well as extremely time-consuming and would ultimately impair the performance comparison. It is thus assumed that the true number of clusters is a given parameter for each method.

#### Assessing the performance

To compare a partition produced by any of the clustering methods with the original method, the Adjusted Rand Index (ARI)^20^ was used. The ARI provides an agreement score between the two partitions, ranging from 0 (complete disagreement) to 1 (complete agreement). Since the clustering methods are applied to 1000 repetitions by design, the resulting ARI are summarized by classical univariate statistics, and graphically represented by boxplots.

### Application on a real-life dataset: the EPHESUS study

The EPHESUS study^4^ is a randomized multicenter double-blind placebo controlled clinical trial, conducted on 6632 patients having a recent acute Myocardial Infarction (MI) and a Left Ventricular Ejection Fraction (LVEF) lower than 40%, with heart failure or diabetes. The treated group, which received eplerenone, had significantly lower mortality and hospitalization rates. This study has been used previously by our group as a first approach of clustering for clinical trial data, using the latent class analysis (LCA) method^16^.

The clustering techniques presented above were used to partition the patients of the EPHESUS study on 16 clinical features recorded at baseline. The six continuous variables consisted of age (AGE), body mass index (BMI), left ventricular ejection fraction (LVEF), estimated glomerular filtration rate (GFR), potassium (K), and sodium (NA). The categorical variables consisted of 9 binary ones: gender (SEX), anemia, hypertension (HTN), diabetes mellitus (DIAB), chronic obstructive pulmonary disease (COPD), atrial fibrillation (AFIB), previous stroke, angina pectoris, and percutaneous coronary intervention or coronary-artery bypass grafting (PCI_CABG), and one polytomous one: smoking status (SMK).

For the purpose of this analysis, the number of clusters of subjects was set to 4 for all the algorithms tested.

These clusters were subsequently analyzed from three perspectives:

- Prognostic ability: Does the survival differ significantly between groups?
- Predictive ability: Does the treatment effect differ significantly between groups?
- Characteristics of the cluster: which key baseline variables best discriminate the clusters?

In order to analyze these dimensions, Kaplan–Meier representations of the risk as well as Cox models with interaction were used. The C-index was used to quantify the ability of the model to predict the primary outcome. The mean treatment effects in each group and their respective 95% confidence interval are presented via forest plots. Finally, radar-charts and absolute standardized mean differences (ASMD) were used to quantify the discriminative influence of the variables used in the clustering.

All methods used with simulated clusters were also used with the EPHESUS data. For the LCA method, the continuous variables were discretized according to the method described in Ferreira et al^16^.

## Results

### Simulated datasets

#### Impact of population properties

**Figure 1:**
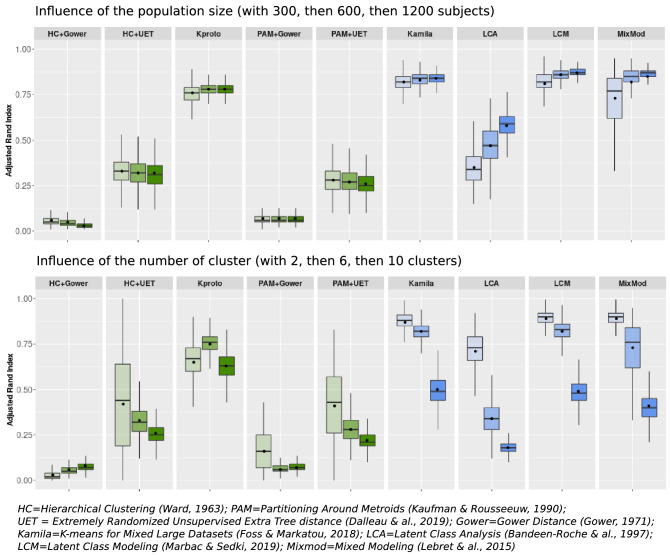
Influence of population size and number of clusters on clustering performance in simulation studies. This figure title was generated using R (R: A Language and Environment for Statistical Computing, R Core Team, R Foundation for Statistical Computing, Vienna, Austria, 2020, https://www.R-project.org).

Figure 1 shows the impact of population size (left panel) and the number of clusters (right panel) on the mean ARI calculated with each clustering method (scenarios 1 and 2 respectively in Table 2). The first five sets of boxplots (in green) are from the distance-based methods, while the four remaining sets of boxplots (in blue) are from the model-based techniques.

An increase in the number of subjects (Fig. 1, left panel) appeared to only positively impact LCA and Mixmod, two model-based methods. Meanwhile, Kamila, Kproto and LCM exhibited a constant good performance irrespective of population size, surpassing the ARIs achieved with other methods.

When increasing the number of clusters up to 10 (Fig. 1, right panel), the mean ARIs dropped for all methods. K-prototypes outperformed all other techniques when the number of clusters was maximum (10 in the present instance). LCM and Kamila displayed the shortest boxplot size, indicating the most reliable results over replications.

For both population size and number of clusters scenarios, the model-based methods and K-prototypes attained higher ARIs than other distance-based methods. Specifically, the Gower distance yielded the worst results when combined with either HC or PAM methods.

#### Impact of the characteristics of the variables

**Figure 2:**
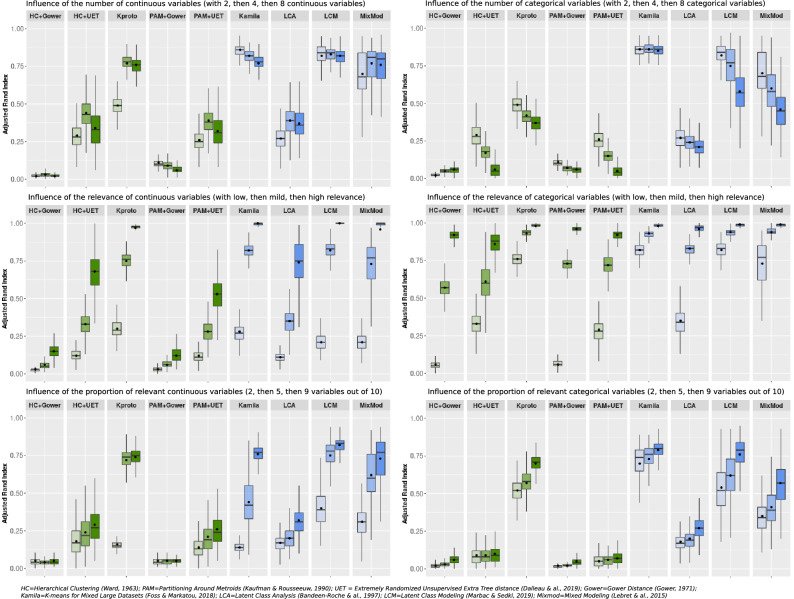
Influence of characteristics of continuous and categorical variables on clustering performance in simulation studies. Top panels: scenario 3 applied to continuous (left) and categorical (right) variables; middle panels: scenarios 4 (left) and 5 (right) ; bottom panels : scenarios 6 (left) and 7 (right)—as defined in Table 2. This figure title was generated using R (R: A Language and Environment for Statistical Computing, R Core Team, R Foundation for Statistical Computing, Vienna, Austria, 2020, https://www.R-project.org).

Figure 2 shows the influence of the characteristics of the variables on clustering performance.

When increasing the number of continuous variables with a constant number of categorical variables [ratio numeric versus categorical 1:2, 1:1 and 2:1)], the ARI of K-prototypes increased, while the ARIs of Kamila, LCM and Mixmod were constantly high. In contrast, when the proportion of categorical variables increased symmetrically, the ARIs of all methods decreased except for Kamila, which maintained a low variance and a satisfactory mean ARI in all cases.

The impact of the respective relevance of continuous and categorical variables is illustrated in the middle panels in Fig. 2. As expected, the mean ARIs increased with the relevance of the variables. Indeed, clustering becomes an easier task when all variables are more relevant.

The impact of varying the proportion of relevant variables (2/10, 5/10 and 9/10), is illustrated in the bottom panels of Fig. 2. Kamila, LCM, Mixmod and K-prototypes exhibited rising ARIs with the reduction in noise.

We used statistical tests to compare ARIs within distance-based methods, within model-based methods and between models-based and distance-based methods. All of these numerous comparisons (96 tests) retrieved p values < 0.0001 (Supplementary Table 1).

Importantly, runtimes differed across methods as reported in Supplementary Table 2 for scenarios 1 and 2. Yet none of them exceeded 20 s.

In summary, distance-based methods (except the K-prototypes) displayed low ARIs in this simulation framework. The lowest ARIs were observed for Gower’s distance either with PAM or hierarchical clustering. In contrast, Kamila and LCM displayed the highest and most stable ARI overall in the vast majority of the scenarios presented herein.

### Application to real-life data: the EPHESUS clinical trial

**Figure 3:**
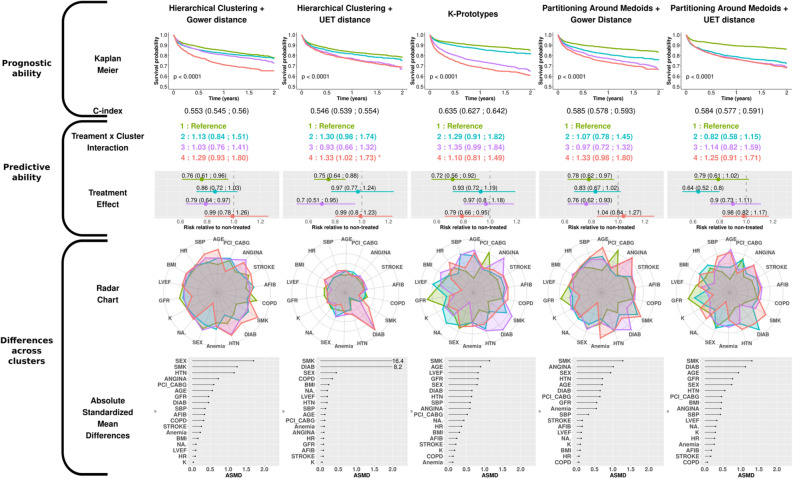
Results obtained with the distance-based clustering algorithms in the EPHESUS dataset. This figure title was generated using R (R: A Language and Environment for Statistical Computing, R Core Team, R Foundation for Statistical Computing, Vienna, Austria, 2020, https://www.R-project.org).

**Figure 4:**
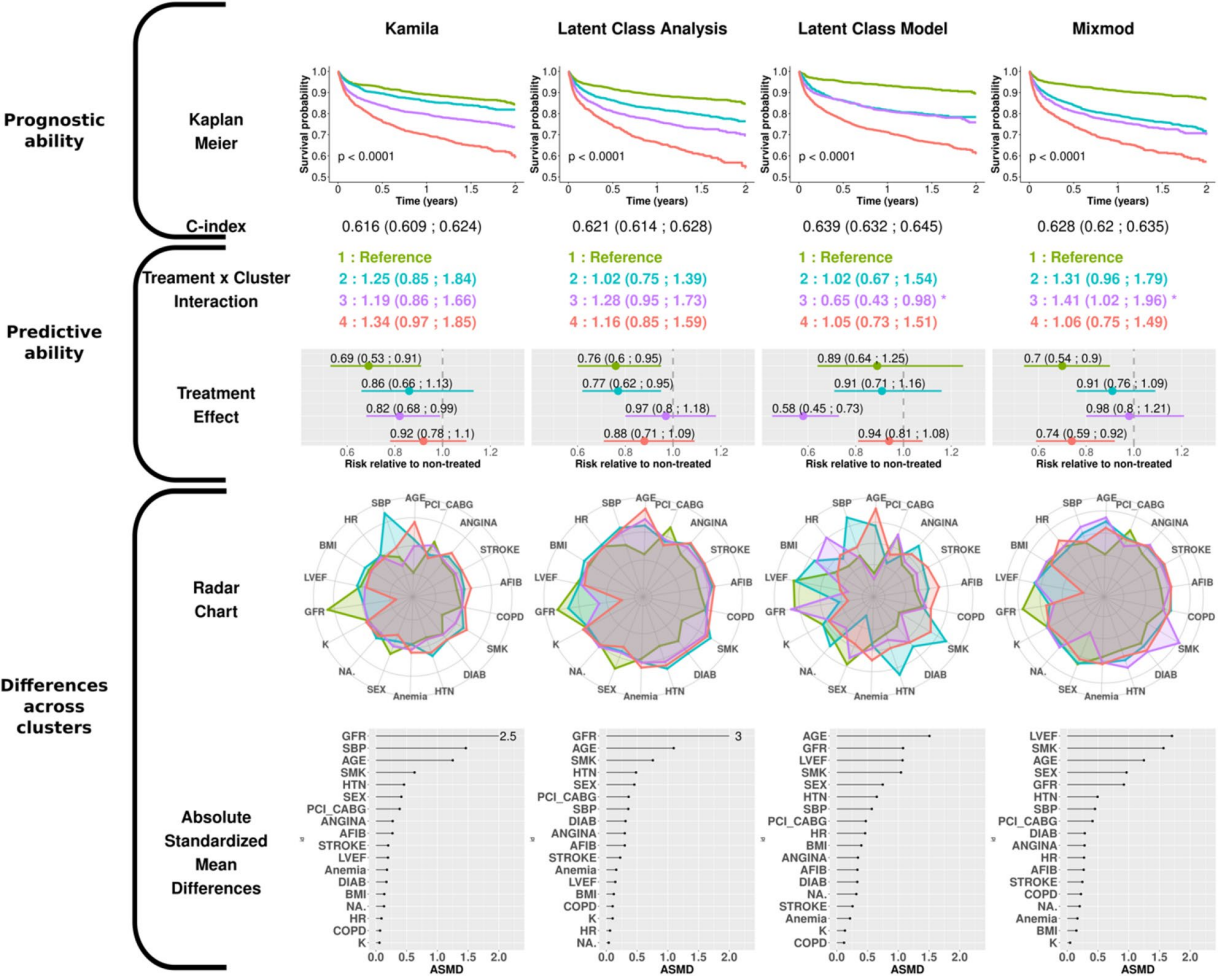
Results obtained with the model-based clustering algorithms in the EPHESUS dataset. Radar charts were created from the means (per, by) group on scaled values (dichotomous and ordinal variables were coded numerically for convenience). The dot charts show the average Absolute Standardized Mean Difference (ASMD) over all clusters. This figure title was generated using R (R: A Language and Environment for Statistical Computing, R Core Team, R Foundation for Statistical Computing, Vienna, Austria, 2020, https://www.R-project.org).

The results obtained with distance-based and model-based clustering methods on the EPHESUS dataset are respectively presented in Figs. 3 and 4.

The clusters are numbered from 1 to 4 according to the ascendant overall risk as estimated by the Kaplan–Meier curve. Thus, regardless of the method used, group 1 (green) had the lowest risk of hospitalization or death related to heart failure, while group 4 (red) had the highest risk.

### Prognostic approach

When considering the survival rate of the subjects via the Kaplan–Meier curves, the K-prototypes method was the only distance-based method that distinguished four groups with noteworthy survival differences together with a C-index > 0.60. For model-based methods, LCA distinguished four well-separated survival patterns, which confirmed the findings of Ferreira et al.^16^. Meanwhile, the LCM algorithm, which had the highest C-index of all methods, identified only 3 distinct survival patterns (the 2nd and 3rd clusters virtually displaying identical survival over time).

### Predictive approach

To quantify the treatment effect across clusters, Cox models were constructed with treatment, cluster and respective interaction as covariates. For distance-based approaches, only group n°4 in HC + UET experienced less treatment benefit relative to group 1 (reference ; 0.99 [0.80;1.20] versus 0.75 [0.64;0.88]) (Second row, Fig. 3).

In the model-based results, significant interactions were identified with LCM and Mixmod. The 3rd cluster identified in LCM had the highest risk reduction observed in our analysis (0.58 [0.45; 0.73]) (second row, Fig. 4), whereas the 3rd cluster identified with MixMod showed less treatment benefit than the reference (0.98 [0.8:1.21]).

### Cluster characteristics approach

The radar charts and dot charts depicted in Figs. 3 and 4 highlight the between-cluster differences in the variables used in the algorithms.

For all methods, both gender and smoking status appeared in the top 5 ASMD (Absolute Standardized Mean Differences). The K-prototypes and the PAM + UET methods yielded the most distinct radar shapes among distance-based clustering methods. Interestingly, the partition provided by the HC + UET was highly driven by two well-known risk factors: smoking status and history of diabetes (ASMD of 16.4 and 8.2 respectively). The groups exhibiting the highest proportion of these factors were also those most at risk, given the overall survival probability. Considering the model-based method, only the Latent Class Model method had very distinct cluster profiles on the radar chart. Age, glomerular filtration rate (GFR) and smoking status were in the top 5 highest ASMD for all model-based methods.

Overall, on the EPHESUS trial data, the K-prototypes and the LCM methods identified clusters with notable differences in characteristics and prognosis although only the LCM method identified a subgroup with substantial treatment effect.

## Discussion

The present findings highlight substantial differences in clustering performance (as measured by ARI on simulated data) between the various methods tested; these methods cannot consequently be considered as interchangeable in the setting of heterogeneous data. Overall, our simulations demonstrate the dominance of K-prototypes, Kamila and LCM over all other methods. In addition, the classical methods using dissimilarity matrices such as Partitioning Around Medoids and Hierarchical Clustering generally performed poorly in comparison with model-based methods. Furthermore, when applying the clustering methods to a real-life clinical dataset, LCM yielded the most promising results, in that it (1) featured the most striking differences in clinical profiles across clusters, (2) exhibited the best prognostic performance of all clustering methods (highest C-index) and (3) identified a subgroup of patients with substantial treatment effect (HR < 0.6).

### Large differences in performance of clustering methods in the benchmark study

Complex situations in cluster analysis have already been emphasized (e.g. in the scikit-learn documentation, https://scikit-learn.org/stable/modules/clustering.html). Yet, in our analysis, as shown in Supplementary Fig. 2, the clusters are relatively well defined, and have “classical” shapes as illustrated by the t-SNE analysis. This suggests that the complexity/difficulty of the clustering is emerging from the heterogeneity of the data rather than the “shape” of the clusters.

The satisfactory performance of LCM and Kamila was partly expected since the simulated data matched their assumptions (better grasp of data heterogeneity). However, Kamila appeared to better tackle the high imbalance between continuous and categorical data than any other method.

Although Mixmod featured a good mean ARI, its great variability (as assessed by the width of the box plots) calls into question the reliability of this algorithm in a given experiment. This level of performance variability may be explained by the choice of the mixture model, which was the least constrained of the 40 available models. Indeed, it allowed different cluster sizes, volumes, shapes, as well as different parameter sets according to cluster, variable and levels. Given all the parameters to be estimated, the number of runs (only one by default) and iterations (200 by default) may not have been sufficient to reach the global minima. Nevertheless, increasing the number of runs and iterations would have led to an impractical computation time, in this context of simulations.

The K-prototypes technique is an interesting case, being the only efficient distance-based method in this benchmark. Although conceptually similar to PAM, its weighted combination of distances may be the key to its success. However, its performance may be greatly diminished on unfavorable datasets with continuous variables of low relevance, (as illustrated in Fig. 2).

The under-performance of LCA may be due to the choice of the cut-points to discretize the continuous variables^21^. Indeed, the cut-point was determined automatically such that three balanced classes arose from each continuous variable, which is not likely to reflect the underlying partition. Nevertheless, this approach represents the routine use of LCA, which is a popular clustering approach.

### Choosing the most appropriate clustering methods

With the present results in mind, the following points could help researchers in choosing adequate clustering methods in the setting of heterogeneous data.

- If the dataset is assumed to stem from a normal-multinomial distribution, model-based methods should logically be preferred. If supplementary assumptions are made relative to cluster size, orientation, volume and levels, a sound option could be to use the corresponding model offered by the Mixmod method. However, using LCM (VarSelLCM package) will probably be sufficient, including additional extra features such as a selection of covariates, handling of missing data, and selection of the number of clusters.
- When the normal-multinomial assumption does not hold, the use of Kamila seemingly appears as a safer choice as opposed to LCM or Mixmod. The K-prototypes method could also be a good alternative, as shown here for datasets with scaled continuous variables (or when continuous variables do not need scaling because they have the same magnitude), and for datasets in which each level of categorical variables is equally represented.
- From a time-efficiency standpoint, the Kamila method offers the best performance when dealing with large datasets (thousands of observations, dozens of variables). Indeed, the method has been specifically implemented to work in a Big-Data setting by taking advantage of the scalability of its algorithm. For other model-based methods such as LCM and Mixmod, the computation time depends on the complexity of the selected model, the number of iterations and the additional features.

### Perspectives

The present analysis aimed to provide some guidance in the choice of a clustering method for heterogeneous data. Given the growing access to multiple data sources, it has become crucial to be able to manage all types of variables through recent advances in statistics and machine-learning. While a few clustering tools for mixed data have been investigated in the present analysis, many more have been developed for this purpose. Nonetheless, this abundance of techniques may confuse end users, since they generally ignore the performance of these techniques relative to each other. As noted by Ahmad and Khan^1^, this mainly stems from the fact that the methods are often evaluated on a restricted number of datasets which cannot be extrapolated to every usage. For example, the popular “Heart-Cleveland” dataset has an arbitrary target variable, while the “Australian Credit” dataset has only a binary target. The generated simulated datasets herein were hence based on a combination of the Qiu and Joe’s^17^ method and a simple home-made stratified sampling.

The benchmark itself, although conducted over a limited selection of methods, revealed a huge performance gap observed between “popular” algorithms (such as Hierarchical Clustering and PAM) and more sophisticated methods (such as Kamila or LCM) in all tested scenarios. However, this does not imply that the former will never be useful, or that the latter will always perform better. For instance, in a setting where groups are nested circles, Kamila will fail to identify the clusters, whereas a hierarchical algorithm with a single linkage aggregation method will easily complete the task.

Even if a clustering technique yields excellent results, its potential is nonetheless ineffective if such technique cannot be implemented in current popular software packages. Indeed, efficiently translating an algorithm into a program is an arduous task for most users of such tools. Of even greater importance is that the quality of the implementation directly determines the computation time. Despite the increased performance of current computers, the clustering time can rapidly become overwhelming, especially when multiple runs are required to determine the number of clusters, or in selecting variables such as in the LCM algorithm. This aspect should not be underestimated as the datasets can quickly reach huge proportions in a big-data context. As already stated above, the Kamila algorithm is particularly well suited for a big-data setting.

According to Hennig^22^, multiple relevant partitions can be found in a population, just as a deck of cards can be clustered according to colors, shapes, values or faces. Consequently, cluster analysis can be considered as successful only if the partition makes sense for the practitioner. Therefore, the involvement of field specialists in the clustering process is essential to determine the question of interest as well as a suitable knowledge of the relevant variables or the number of clusters. Despite the immense progress enabled by artificial intelligence in recent years, human experience and intuition remain the best judge in cluster analysis. That being said, it is very likely that useful clinical information will arise only from clustering algorithms displaying good intrinsic performance. In keeping with the latter, the results of the present benchmark analysis could strengthen the collaboration of data-analysts, clinical researchers and physicians by using the most appropriate machine-learning tools. Importantly, our results provide guidance on how to use all available (heterogeneous) clinical, biological and imaging data for clustering analysis in clinical research cohorts. UET as a stochastic–based method for computing pairwise distances while avoiding the burden of data preparation is particularly relevant both for large heterogeneous datasets (with thousands of variables) and structures^6^. Conversely, its underperformance could be related to the simulated datasets using normal distributions for continuous variables, which are a better fit with model-based methods. Nonetheless, a number of continuous variables often follow a normal distribution in clinical research.

### Limitations

Although the *genRandomClust* function allows unbalanced cluster sizes and outliers, these features were not used herein due to lack of time. The impact of these characteristics would be worth investigating in subsequent studies. In addition, scenarios with non-normal continuous variables may yield different results and should be tested in subsequent studies. However, our simulation study already covers a number of scenarios which could already be useful in a range of clinical studies.

An important limitation of our work is the use of the Hamming distance for the categorical data in the distance-based algorithm implemented in R software (as shown in Table 1). The use of other approaches could have resulted in largely different clustering performance. More advanced approaches, such as the novel clustering algorithm developed by Hautamaki et al.^23^, based on local search for its objective function using entropy, could provide clustering quality comparable to the ones obtained with model-based approaches in our analysis. However, to our knowledge, such algorithms are not yet incorporated in “ready-to-use” dedicated software packages, easily usable by non-expert teams.

## Conclusion

The results from the present simulation study focused on ”ready-to-use” tools from R and suggest that model-based tools (p.e. the Kamila and LCM packages implemented in R) usually perform better than distance-based tools (except K-prototypes packages implemented in R) in the setting of heterogeneous data such as clinical research datasets including both numeric and categorical variables. The present results suggest that model-based tools that are currently readily available to biology and clinical researchers can be useful practical solutions for performing clustering in situations involving heterogeneous data. Future work should continue this effort for benchmarking "ready-to-use" clustering tools on mixed data, possibly using our simulated datasets (available on http://mbi.loria.fr/clustering-of-mixed-data/). Eventually, benchmarking platforms of mixed data should arise, which would make it possible to test new tools in the future and compare them with previously benchmarked tools. We believe this effort is necessary as only the use of the most relevant approaches could improve our ability to identify clinically relevant subgroups in numerous clinical settings, by feeding efficient clustering algorithms with both clinical and biological data.

## Supplementary Information

Supplementary Information.
