# Supplementary material for: Head-to-head comparison of clustering methods for heterogeneous data: a simulation-driven benchmark
Source: Sci Rep. 2021 Feb 18;11:4202. doi: 10.1038/s41598-021-83340-8 (PMC7892576; doi:10.1038/s41598-021-83340-8)
Supplement: Supplementary file 1 — Supplementary Information. [file 41598_2021_83340_MOESM1_ESM.docx]

**Head-to-head comparison of clustering methods for heterogeneous data**

***A simulation-driven benchmark***

***SUPPLEMENTARY FIGURES AND TABLE***

**Supplementary Figure 1: Introduction of controlled noise in a simulated categorical variable with three levels (A: pink, B: green and C: blue) as performed in the present analysis (left panel: 0% noise, right panel: 20% noise).**


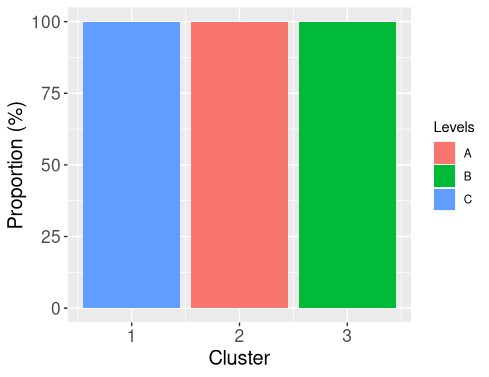

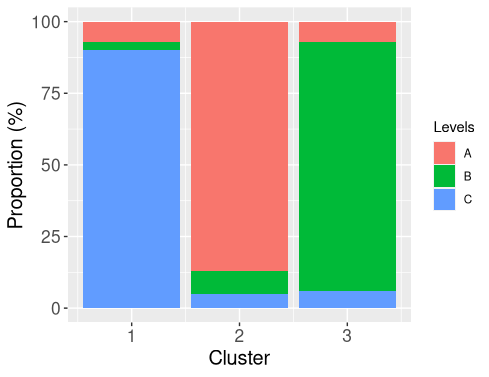


**Supplementary figure 2: 2D projections of numerical variables for datasets created from the simulation scenarios using t-SNE.**

Cluster memberships are denoted by their color.


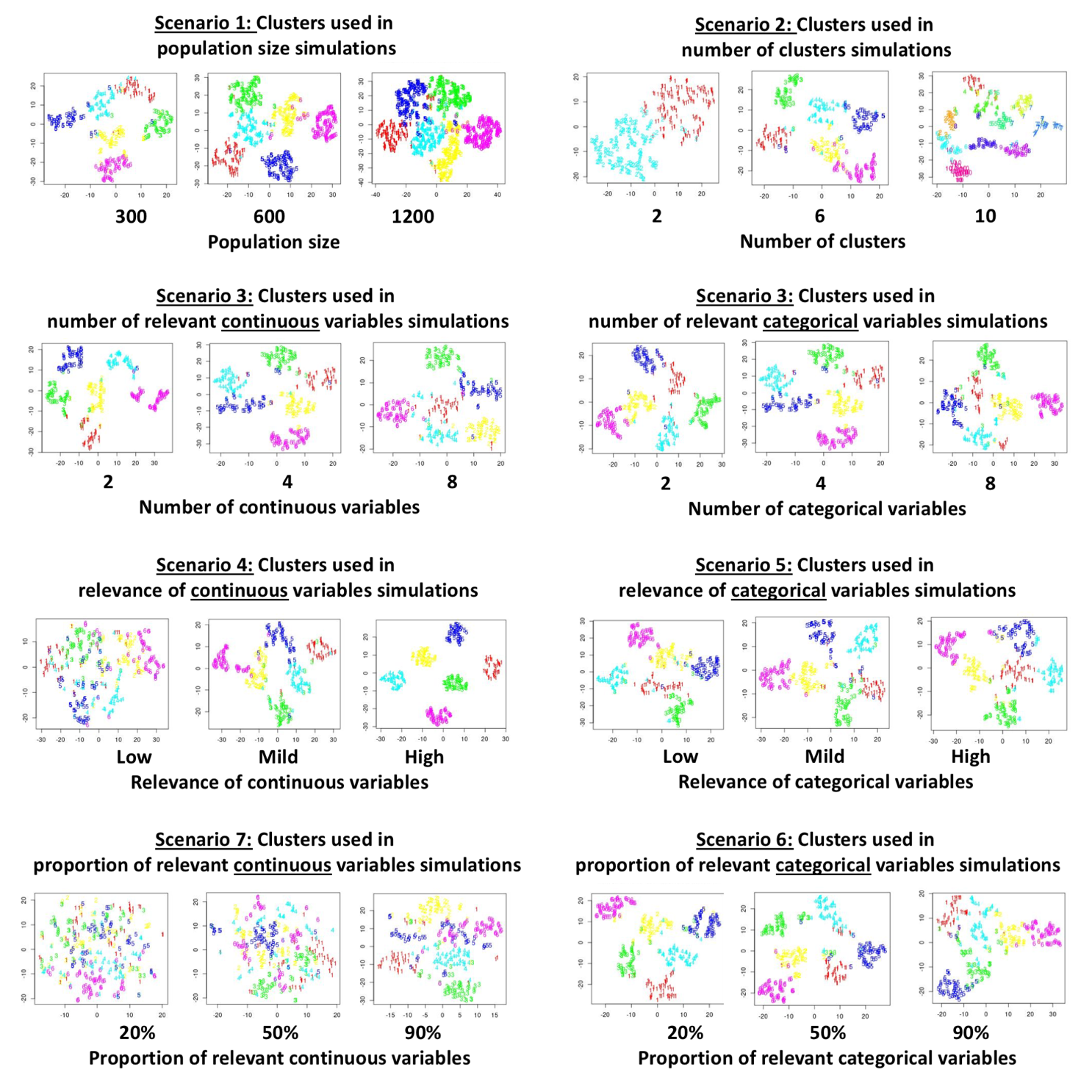


**Supplementary table 1: Comparison of methods using ARI metric for each condition presented in figures 1 and 2**

| **Figure** | **Condition** | | **Overall**  **comparison*** | **Comparison among**  **distance-based**  **methods*** | **Comparison among**  **model-based**  **methods*** | **Comparison**  **model-based vs.**  **distance-based**** |
| --- | --- | --- | --- | --- | --- | --- |
| **1** | **Population size** | **300** | <0.0001 | <0.0001 | <0.0001 | <0.0001 |
| **1** |  | **600** | <0.0001 | <0.0001 | <0.0001 | <0.0001 |
| **1** |  | **1200** | <0.0001 | <0.0001 | <0.0001 | <0.0001 |
| **1** | **Number of clusters** | **2** | <0.0001 | <0.0001 | <0.0001 | <0.0001 |
| **1** |  | **4** | <0.0001 | <0.0001 | <0.0001 | <0.0001 |
| **1** |  | **6** | <0.0001 | <0.0001 | <0.0001 | <0.0001 |
| **2** | **Number of**  **continuous variables** | **2** | <0.0001 | <0.0001 | <0.0001 | <0.0001 |
| **2** |  | **4** | <0.0001 | <0.0001 | <0.0001 | <0.0001 |
| **2** |  | **6** | <0.0001 | <0.0001 | <0.0001 | <0.0001 |
| **2** | **Number of**  **categorical variables** | **2** | <0.0001 | <0.0001 | <0.0001 | <0.0001 |
| **2** |  | **4** | <0.0001 | <0.0001 | <0.0001 | <0.0001 |
| **2** |  | **6** | <0.0001 | <0.0001 | <0.0001 | <0.0001 |
| **2** | **Relevance of**  **continuous variables** | **low** | <0.0001 | <0.0001 | <0.0001 | <0.0001 |
| **2** |  | **mild** | <0.0001 | <0.0001 | <0.0001 | <0.0001 |
| **2** |  | **high** | <0.0001 | <0.0001 | <0.0001 | <0.0001 |
| **2** | **Relevance of**  **categorical variables** | **low** | <0.0001 | <0.0001 | <0.0001 | <0.0001 |
| **2** |  | **mild** | <0.0001 | <0.0001 | <0.0001 | <0.0001 |
| **2** |  | **high** | <0.0001 | <0.0001 | <0.0001 | <0.0001 |
| **2** | **Number of relevant**  **continuous variables** | **2/10** | <0.0001 | <0.0001 | <0.0001 | <0.0001 |
| **2** |  | **5/10** | <0.0001 | <0.0001 | <0.0001 | <0.0001 |
| **2** |  | **9/10** | <0.0001 | <0.0001 | <0.0001 | <0.0001 |
| **2** | **Number of relevant**  **categorical variables** | **2/10** | <0.0001 | <0.0001 | <0.0001 | <0.0001 |
| **2** |  | **5/10** | <0.0001 | <0.0001 | <0.0001 | <0.0001 |
| **2** |  | **9/10** | <0.0001 | <0.0001 | <0.0001 | <0.0001 |

*p-value from Kruskal-Wallis test. **p-value from Wilcoxon test.

**Supplementary table 2: Runtimes of the examined method for the conditions presented in figure 1 (average runtimes are presented in seconds).**

Experiments were run on a computer with an Intel i7-4600U processor running at 2.10 GHz (x4) using 11.1 GiB of RAM, running Linux: Ubuntu 20.04 LTS.

|  | **Population size** | | | **Number of clusters** | | |
| --- | --- | --- | --- | --- | --- | --- |
| **Methods** | **300** | **600** | **1200** | **2** | **6** | **10** |
| **HC+Tradi** | 0.021 | 0.089 | 0.433 | 0.024 | 0.030 | 0.021 |
| **HC+UET** | 0.633 | 2.292 | 7.715 | 0.753 | 0.841 | 0.785 |
| **Kproto** | 5.846 | 9.696 | 13.805 | 1.338 | 5.941 | 9.612 |
| **PAM+Tradi** | 0.071 | 0407 | 1.524 | 0.040 | 0.080 | 0.107 |
| **PAM+UET** | 0.662 | 2.420 | 8.602 | 0.759 | 0.875 | 0.864 |
| **Kamila** | 0.715 | 1.065 | 1.443 | 0.327 | 0.837 | 0.724 |
| **LCA** | 3.611 | 11.437 | 19.894 | 0.947 | 4.201 | 7.778 |
| **LCM** | 4.564 | 7.863 | 14.318 | 0.891 | 4.438 | 10.161 |
| **Mixmod** | 0.265 | 0.167 | 0.309 | 0.182 | 0.080 | 0.177 |
